# Supplementary material for: Endovascular progenitors infiltrate melanomas and differentiate towards a variety of vascular beds promoting tumor metastasis
Source: Nat Commun. 2019 Jan 3;10:18. doi: 10.1038/s41467-018-07961-w (PMC6318267; doi:10.1038/s41467-018-07961-w)
Supplement: Supplementary file 1 — Supplementary Information [file 41467_2018_7961_MOESM1_ESM.pdf]

Supplementary Information

Endovascular progenitors infiltrate melanomas and differentiate towards a variety of vascular beds promoting tumor metastasis

Prudence Donovan[1], Jatin Patel[1], James Dight[1], Ho Yi Wong[1], Seen-Ling Sim[1], Valentine Murigneux[1], Mathias Francois[2], Kiarash Khosrotehrani[1]

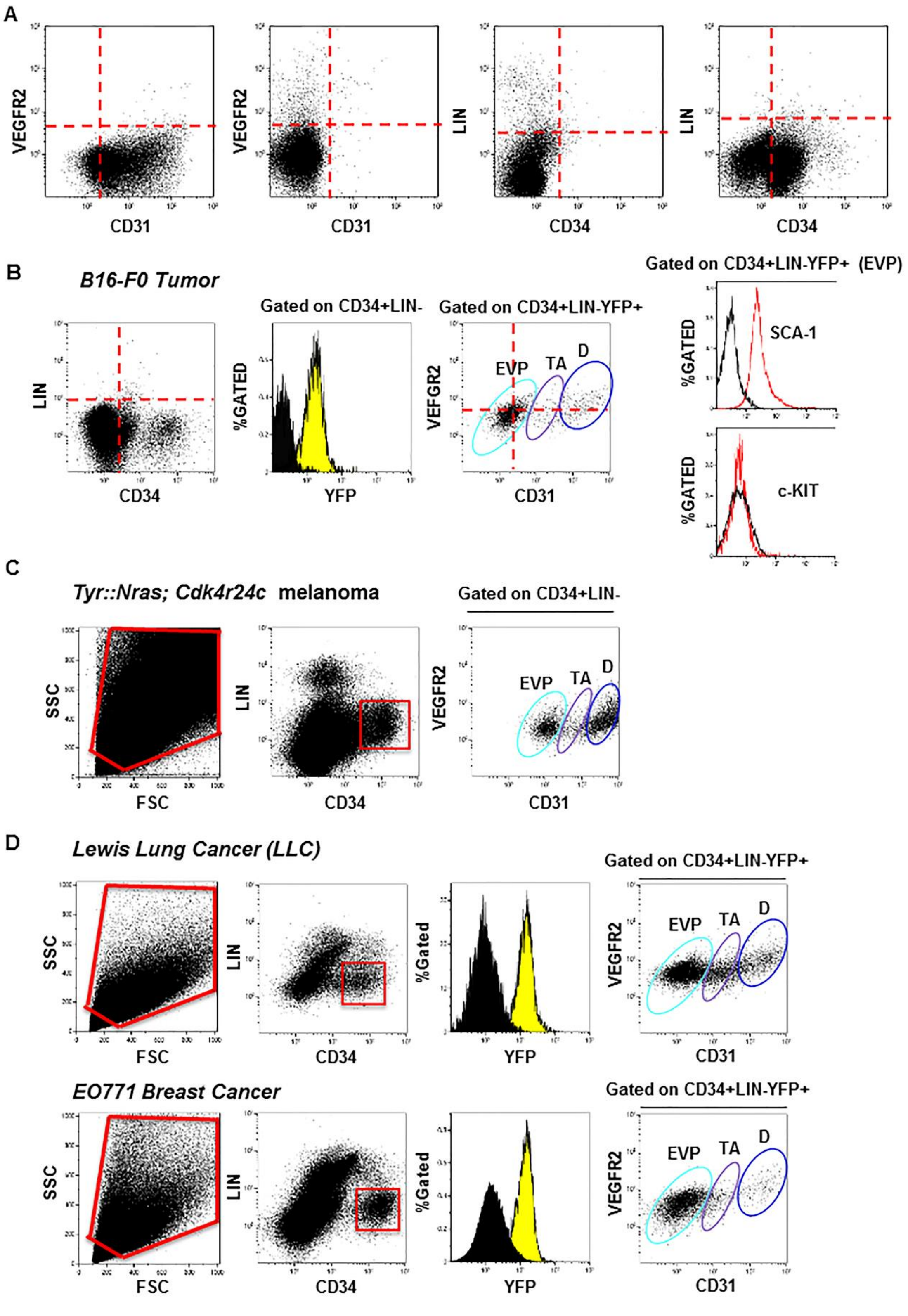

Supplementary Figure 1. Defining the endothelial hierarchy in solid tumor settings. (A) Fluorescence-minus-one (FMO) of the endothelial markers used to determine gating strategy. **(B)** *Cdh5-Cre^{ER} RosaYFP* lineage tracing used to demonstrate that EVP within B16-F0 tumors are Sca-1+ and negative for hematopoietic marker c-KIT (n=3). **(C)** Flow cytometry plots demonstrating the existence of the endothelial hierarchy in spontaneously developed melanomas using the mouse line *Tyr::Nras;*

Cdk4r24c (n=3). **(D)** Flow cytometry plots demonstrating the existence of the endothelial hierarchy in Lewis Lung Carcinoma (n=3) and EO771 Breast Cancer (n=3) tumor models. EVP – Endovascular progenitor; TA- Transit amplifying; D – Definitive differentiated.

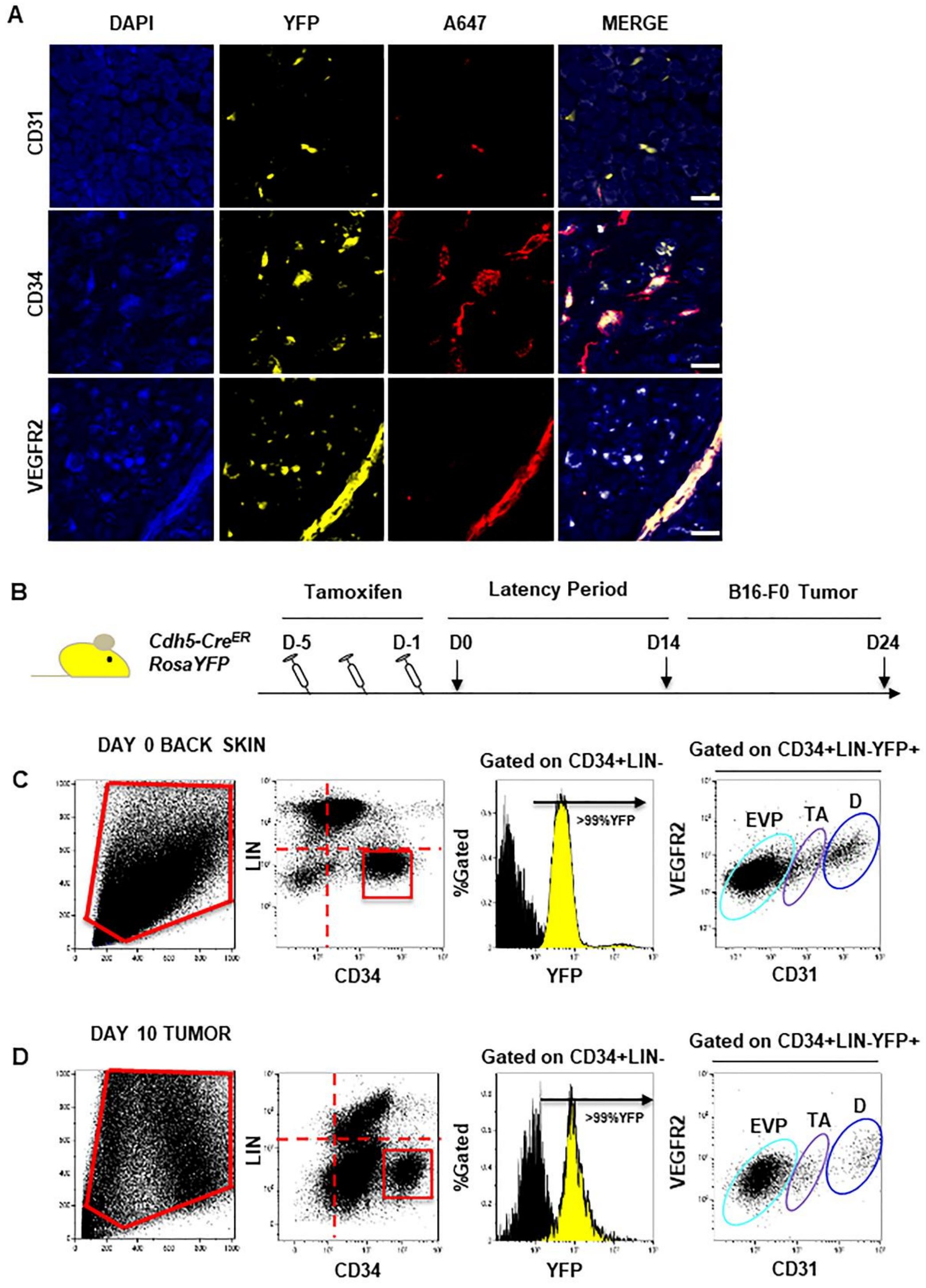

Supplementary Figure 2. Defining the origin of EVP. (A) Representative micrographs of tumor sections taken from *Cdh5-Cre^{ER} RosaYFP* and *Sox18-Cre^{ER} RosaYFP* lineage tracing models demonstrate that the EVP are individual YFP+ foci that are CD34+ but absent for CD31 and VEGFR2. **(B)** Schematic representation of the experimental model used to assess if endothelial dilution was occurring by unlabeled cells after 5 days of tamoxifen administration (n=6). **(C)** Normal back skin was processed and greater than 99% of all LIN-CD34+ cells were YFP+, demonstrating the entire labelling of the vasculature and endothelial hierarchy (n=3). **(D)** The tumors were then collected 10 days later,

or 24 days after their final tamoxifen injection (n=3). Within the tumors greater than 99% of all LIN-CD34+ cells remained YFP+, clarifying that the endothelial hierarchy was only originating from the vasculature and not being diluted by other unlabeled populations. EVP – Endovascular progenitor; TA-Transit amplifying; D – Definitive differentiated.

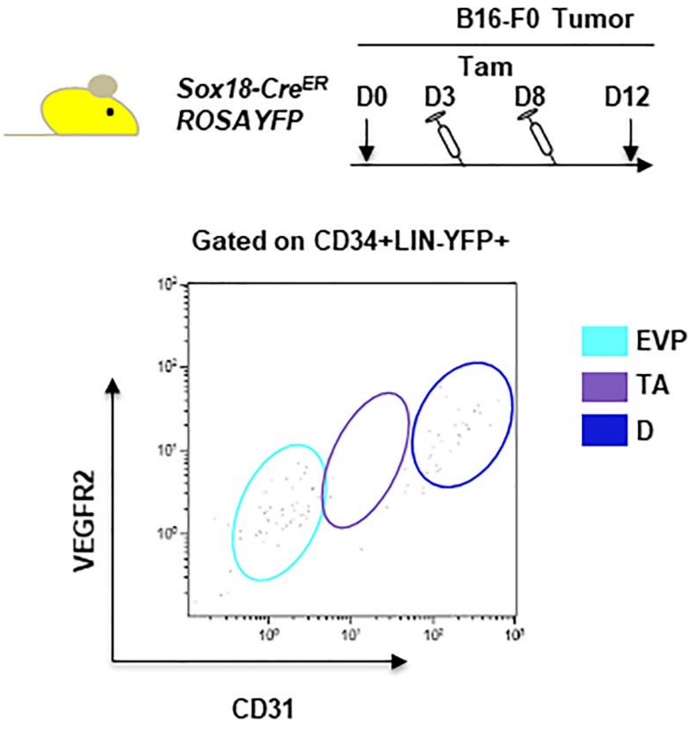

Supplementary Figure 3. Dynamic contribution of EVP over time of tumor growth. Schematic diagram of the *Sox18-Cre*^{ER} *RosaYFP* lineage tracing model employed with B16-F0 melanoma cells injected at day 0 (D0), with mice receiving tamoxifen (Tam) days 3 and 8 (D3, D8) post tumor inoculation (n=3). Flow cytometry plots at D12 post tumor inoculation show YFP+ EVP can be observed, demonstrating the continual migration of EVP during tumor growth. Scale bar represents 50µm. EVP – Endovascular progenitor; TA- Transit amplifying; D – Definitive differentiated.

Term	P-Value	Bonferonni	Benjamini	FDR
**ECM-receptor interaction**	5.59E-16	1.48E-13	1.48E-13	7.33E-13
**Focal adhesion**	1.09E-14	2.91E-12	1.45E-12	1.43E-11
**PI3K-Akt signaling pathway**	6.53E-12	1.74E-09	5.81E-10	8.57E-09
**Protein digestion and absorption**	6.54E-12	1.75E-09	4.36E-10	8.58E-09
**Proteoglycans in cancer**	5.07E-11	1.35E-08	2.71E-09	6.65E-08
**Rap1 signaling pathway**	3.67E-09	9.80E-07	1.63E-07	4.82E-06
**Pathways in cancer**	3.17E-08	8.47E-06	1.21E-06	4.17E-05
**Cytokine-cytokine receptor interaction**	1.16E-07	3.08E-05	3.85E-06	1.52E-04

Supplementary Figure 4. RNA-sequencing data analysis. (A) Pathway analysis (DAVID) shows extra-cellular matrix-receptor interaction and focal adhesion were the top pathways reflecting the activity of EVP cells. **(B-F)** Comparison of key genes from the RNA-sequencing between EVP, TA and D (**$p<0.01$; ***$p<0.001$ vs EVP respectively; Mann Whitney T-Test). EVP – Endovascular progenitor; TA- Transit amplifying; D – Definitive differentiated.

Supplementary Figures 4B-F are on the following pages below.

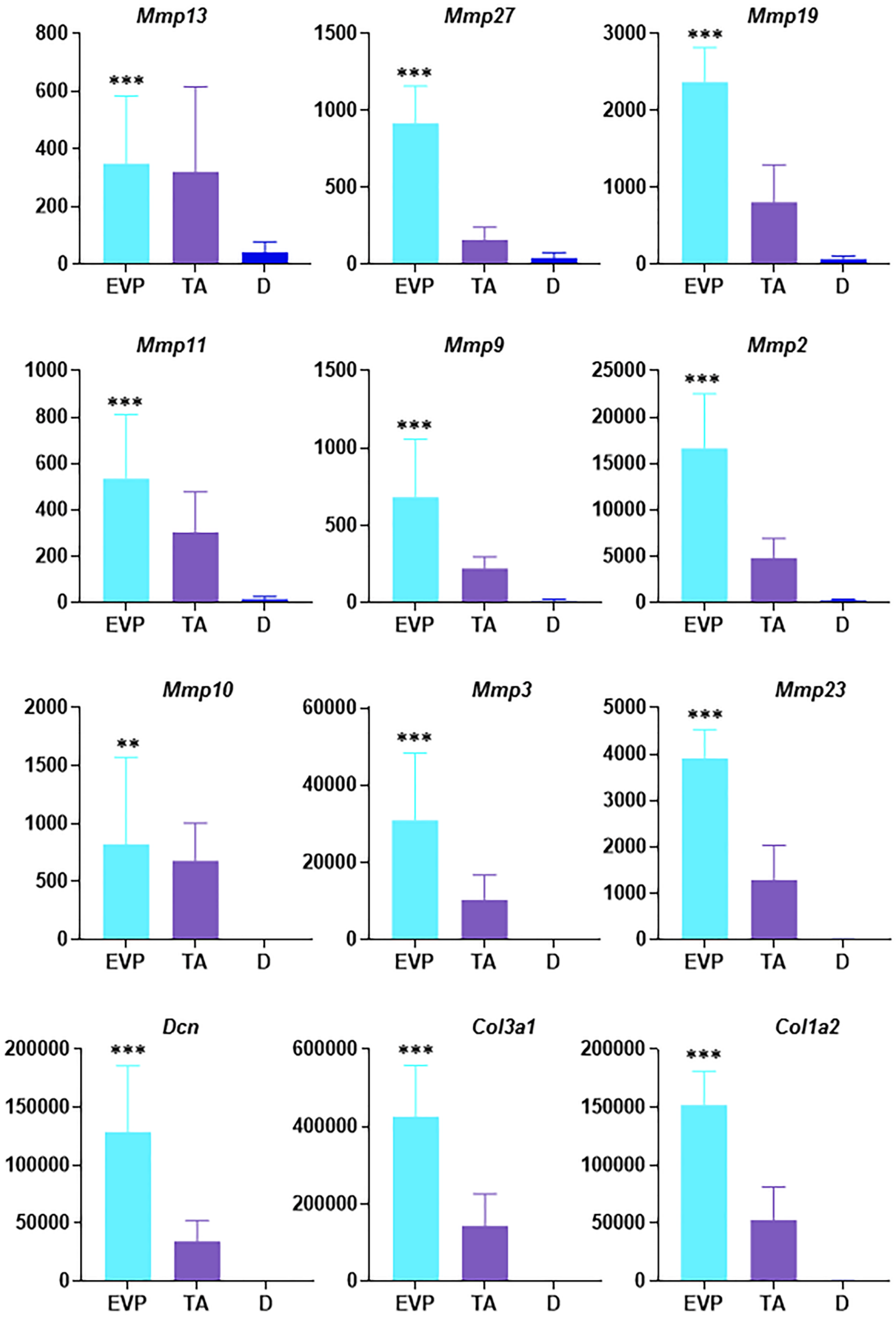

Supplementary Figure 4B

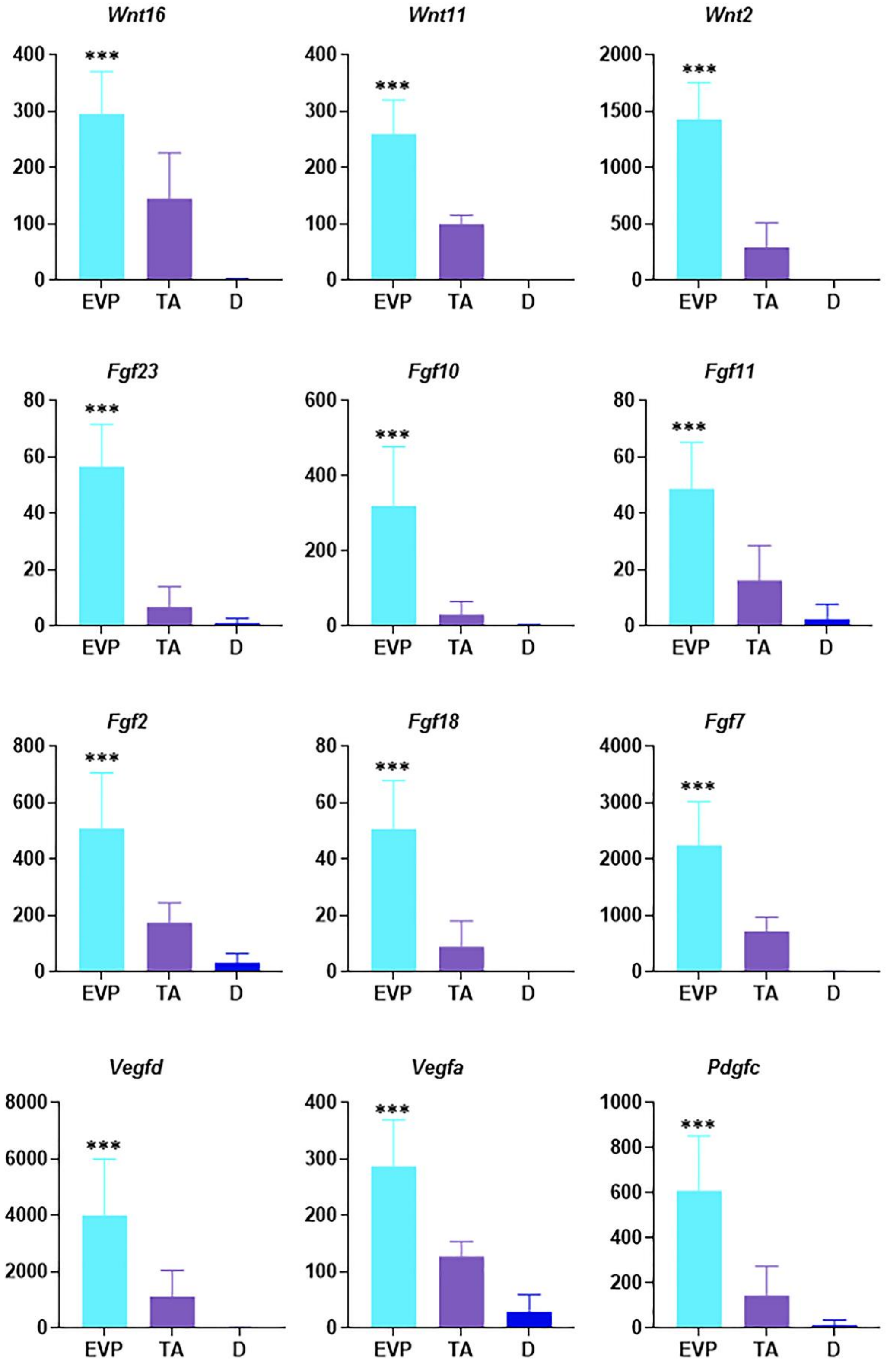

Supplementary Figure 4C

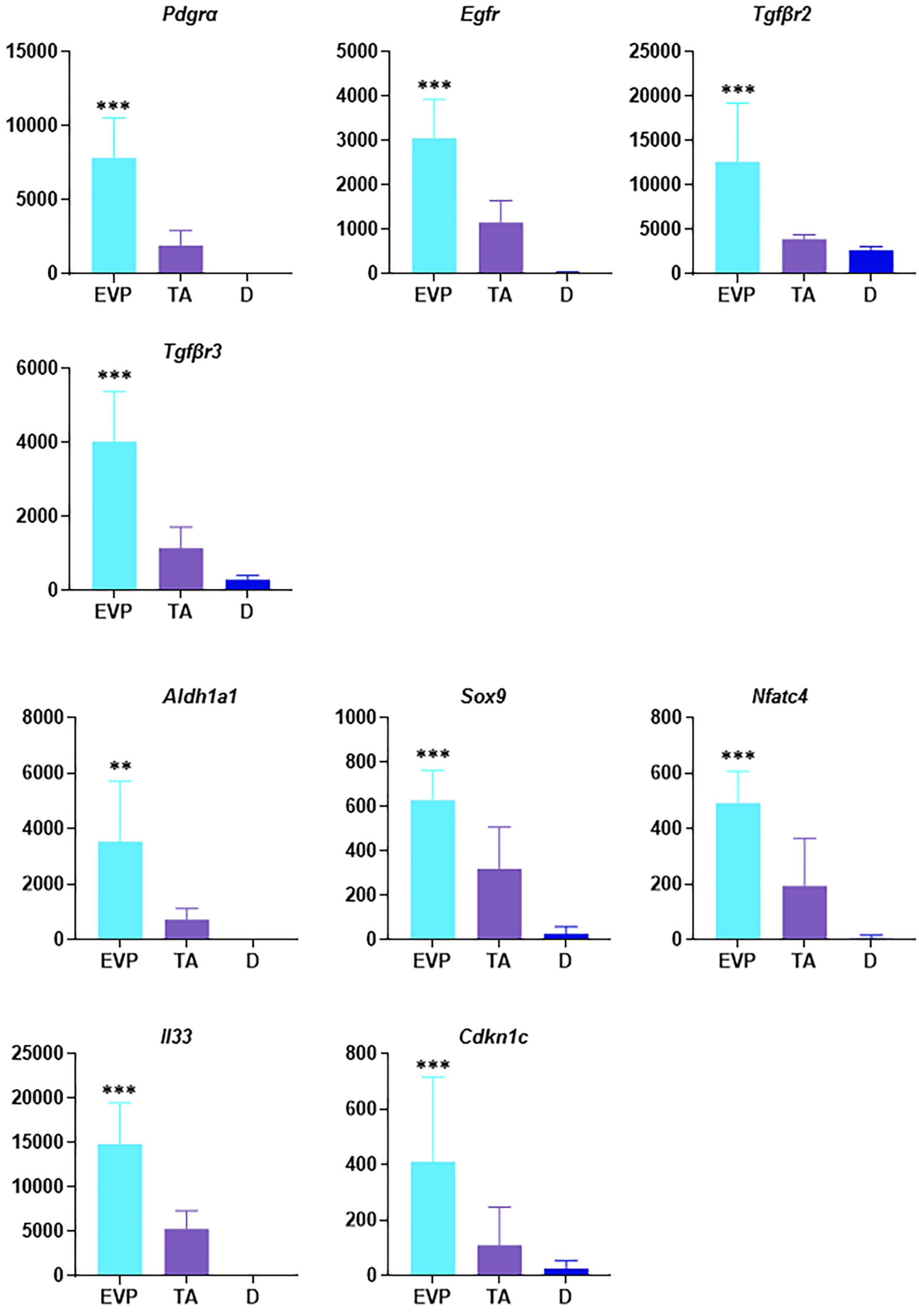

Supplementary Figure 4D

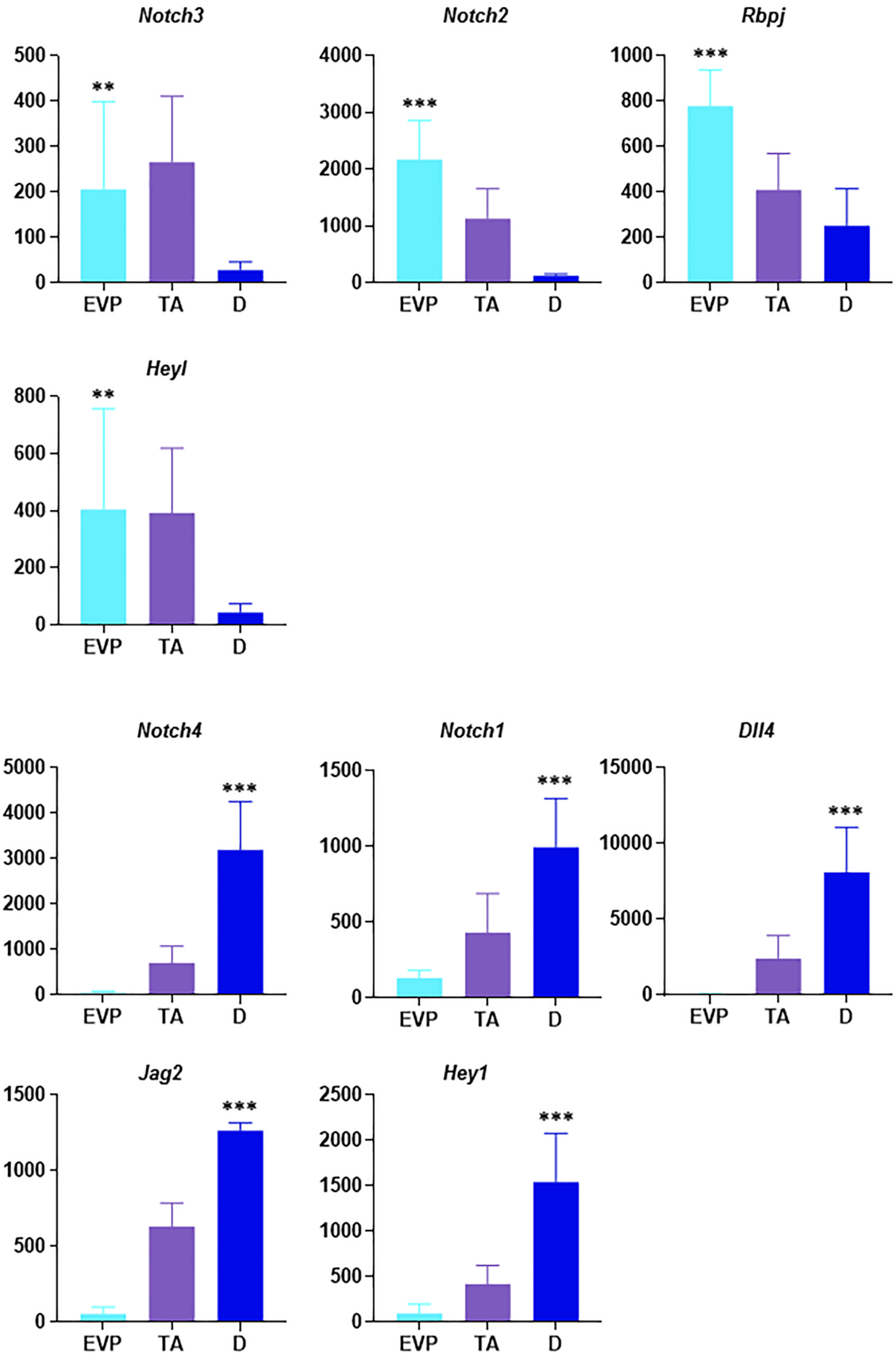

Supplementary Figure 4E

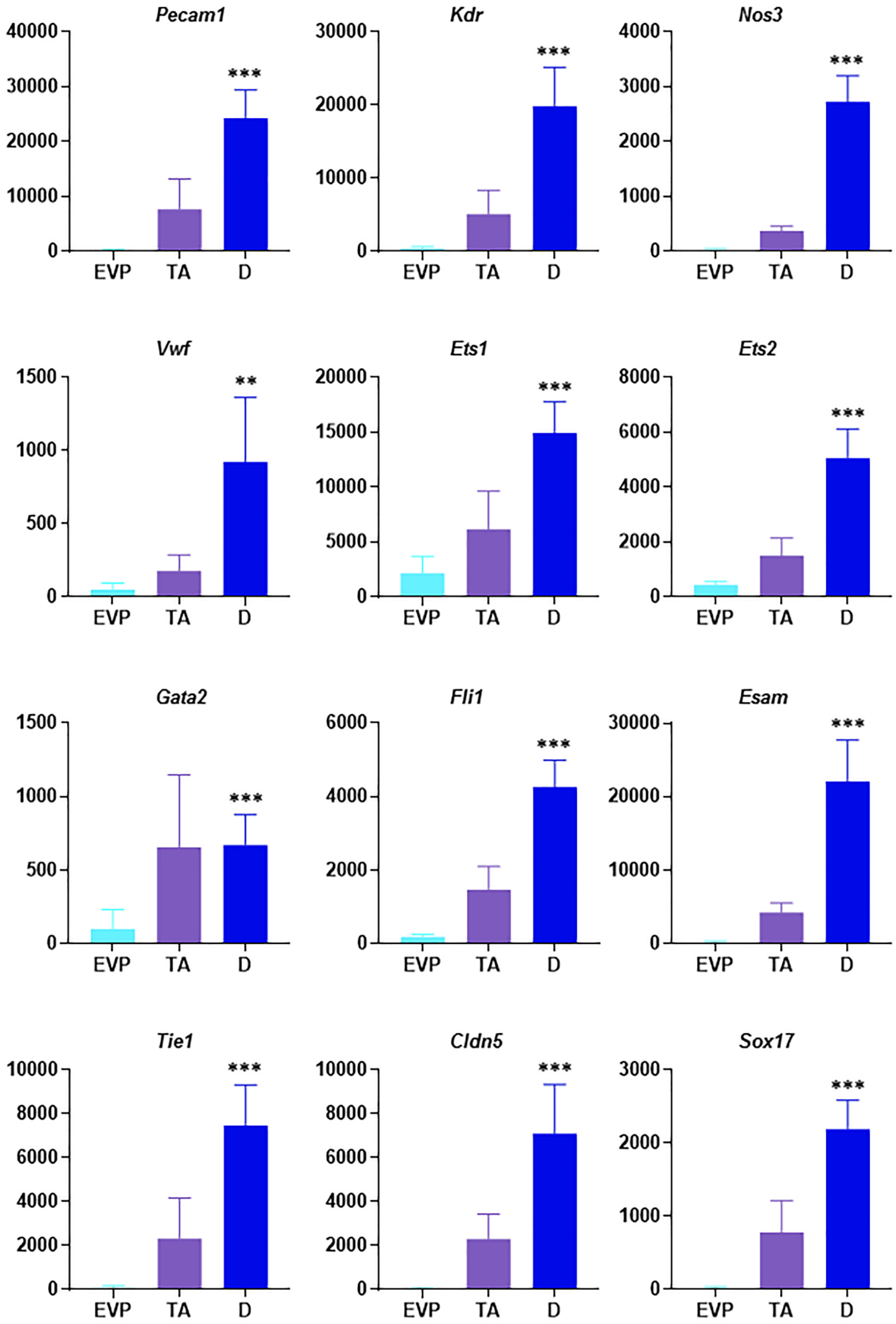

Supplementary Figure 4F

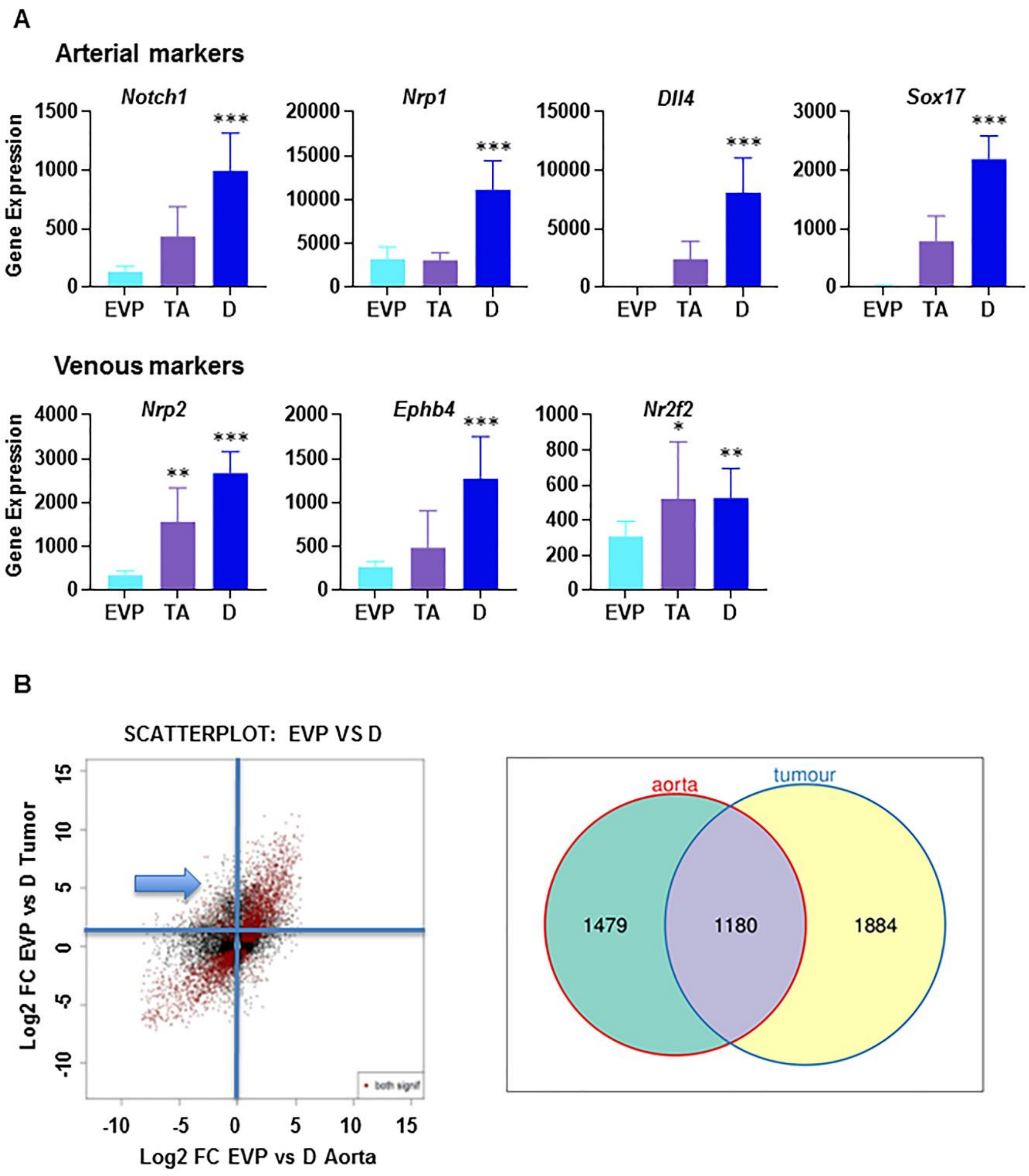

Supplementary Figure 5. RNA-Sequencing data analysis. (A) Expression of arterial and venous genes between EVP, TA and D populations (*p<0.05; **p<0.01; ***p<0.001 vs EVP respectively; Mann-Whitney T-Test). **(B)** Scatter plot demonstrating overlap of gene expression from aorta and tumor EVP RNA-sequencing. Many of the same stem cell genes observed in aorta are upregulated in the EVP in tumors (***p<0.001 vs D). A Venn diagram also indicates the number of genes being differentially regulated in the aorta and tumors. This analysis was limited to significantly upregulated genes with a fold change >2. Results presented as mean +/- SEM. EVP – Endovascular progenitor; TA- Transit amplifying; D – Definitive differentiated.

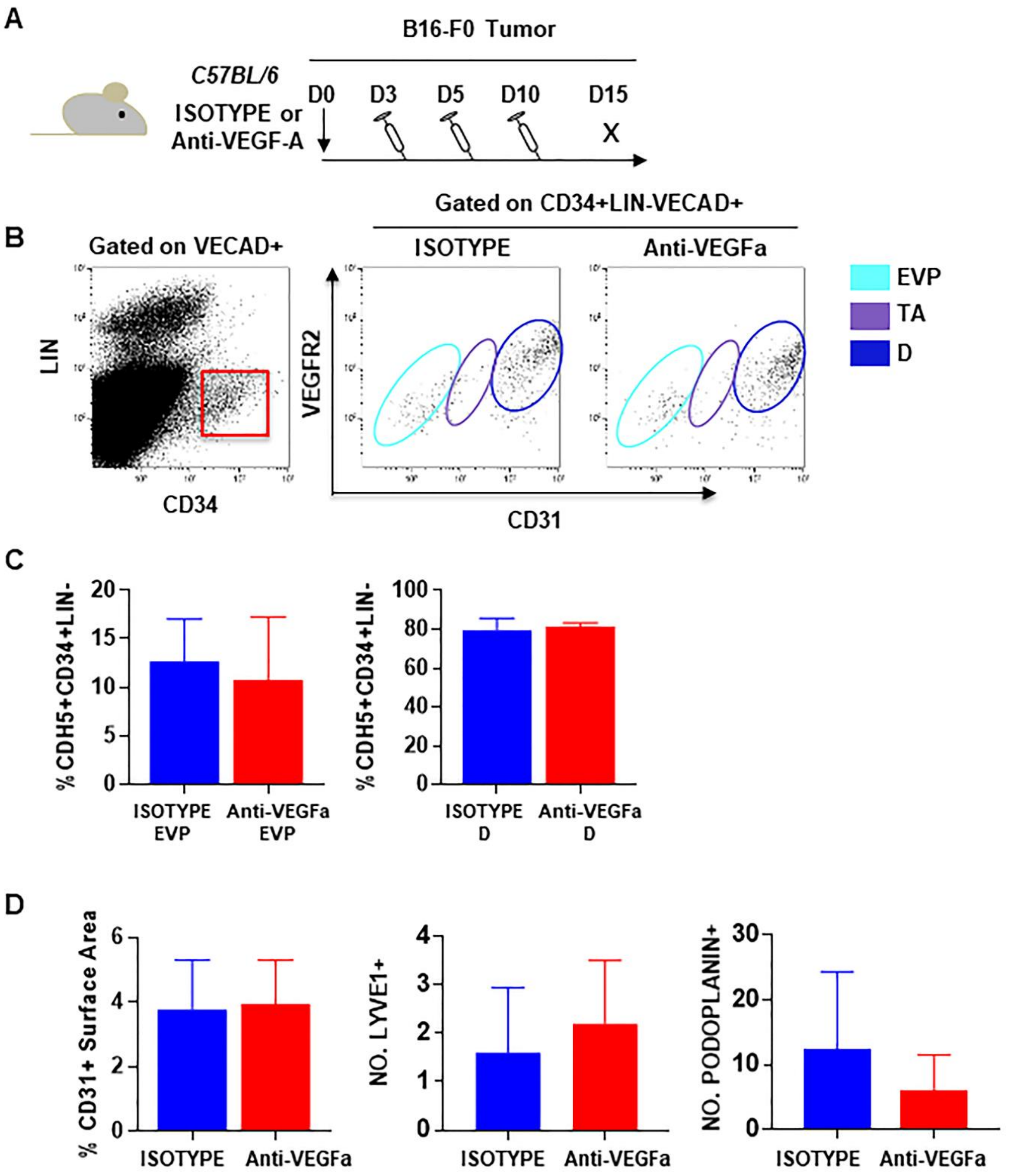

Supplementary Figure 6. Anti-VEGFA treatment. (A) Schematic diagram of C57BL/6 wild-type mice inoculated with B16-F0 melanoma cells injected at day 0 (D0), with mice receiving either isotype or anti-VEGF-A at Days 3, 5 and 10 (D3, D5, D10) (n=5). **(B-C)** Flow cytometry plots demonstrating no change in the numbers of EVP or D present in the tumor between the treatment groups. **(D)** No difference was also observed in percentage CD31+ vessel surface area or number of Lyve1+ or Podoplanin+ vessels (Mann-Whitney T-Test). Results presented as mean +/- SEM. EVP – Endovascular progenitor; TA- Transit amplifying; D – Definitive differentiated.

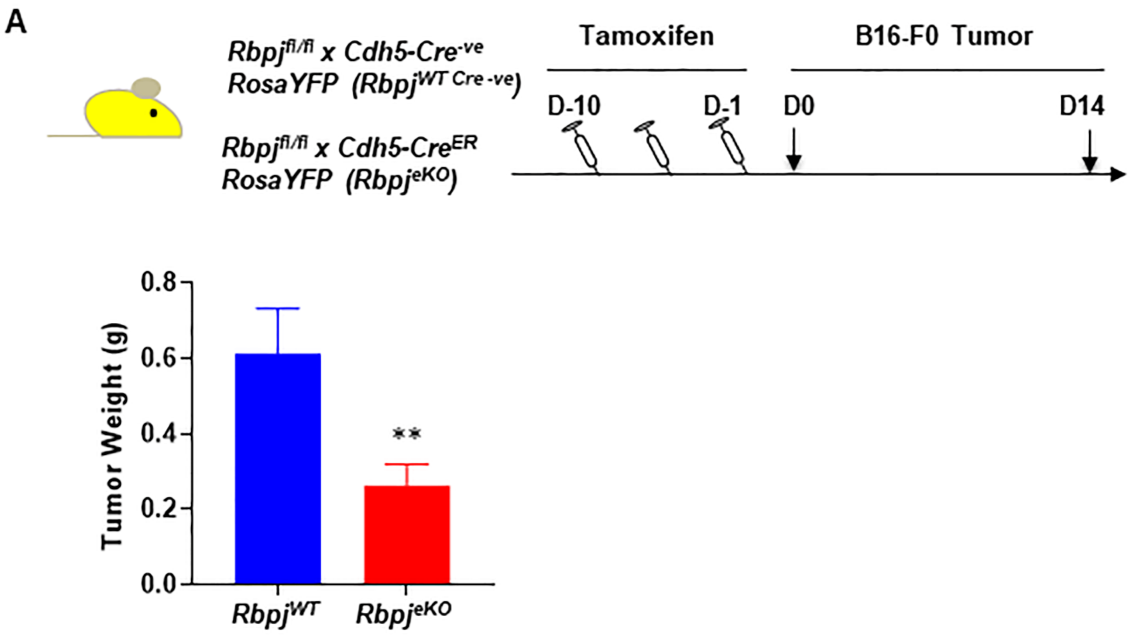

Supplementary Figure 7. Tumor weight after *RBPJ* vascular knockout. Schematic diagram of the *Rbpj*$^{fl/fl}$/*Cdh5-Cre*^{ER} *RosaYFP* (*Rbpj*^{eKO}) and *Rbpj*$^{fl/fl}$ *RosaYFP* (*Rbpj*$^{WT\text{- Cre Negative}}$) lineage tracing model employed, with mice receiving tamoxifen (Tam) for 10 consecutive days prior to tumor inoculation with B16-Fo melanoma cells injected at day 0 (D0). Tumors were collected at D14 and comparison of weight between groups were assessed (**$p<0.01$; Mann-Whitney T-Test; n=5). Results presented as mean +/- SEM.
